# Vasa Is a Potential Germ Cell Marker in Leopard Coral Grouper (*Plectropomus leopardus*)

**DOI:** 10.3390/genes13061077

**Published:** 2022-06-16

**Authors:** Mingyi Wang, Hui Ding, Shaoxuan Wu, Mengya Wang, Cun Wei, Bo Wang, Zhenmin Bao, Jingjie Hu

**Affiliations:** 1 Key Laboratory of Marine Genetics and Breeding (Ministry of Education), College of Marine Life Sciences, Ocean University of China, Qingdao 266003, China; mingyi9802@163.com (M.W.); ding425926225@163.com (H.D.); wushaoxuan@outlook.com (S.W.); mengya0828@163.com (M.W.); zmbao@ouc.edu.cn (Z.B.); 2 Key Laboratory of Tropical Aquatic Germplasm of Hainan Province, Sanya Oceanog Institute, Ocean University of China, Sanya 572000, China; soiweic@ouc.edu.cn; 3 Hainan Yazhou Bay Seed Laboratory, Sanya 572025, China

**Keywords:** teleost, leopard coral grouper, gonad, vasa, germ cell marker

## Abstract

Vasa (*Ddx4*, DEAD box polypeptide 4), an extremely specific marker of germ cells in vivo, is an ATP-dependent RNA helicase that plays an essential role in germ cell development and gametogenesis. However, the expression and function information about this gene in groupers remains lacking. Here, *vasa* homolog termed *Plvasa* was isolated and identified *Plvasa* as a putative germ cell marker in the leopard coral grouper, (*Plectropomus leopardus*). Results indicated that *Plvasa* contained 17 exons in the genomic sequence and 9 conserved motifs of the DEAD-box protein by sequence analysis. The sequence comparison, phylogenetic analyses and synteny analyses showed that *Plvasa* was homologous with other teleosts. Additionally, the expression of *Plvasa* was significantly higher in gonads than in other tissues in adult individuals (*p* < 0.05). Further, the distribution of *Plvasa* revealed that it was only expressed in the germ cells, such as spermatids, germline stem cells and oocytes at different stages, and could not be detected in the somatic cells of gonads. The current study verified that the *Plvasa* gene is a valuable molecular marker of germ cells in leopard coral grouper, which potentially plays an important role in investigating the genesis and development of teleost germ cells.

## 1. Introduction

The understanding of the reproduction mechanism in animals has advanced significantly as a result of the remarkable developments in the molecular, cellular, genetic sciences and other modern biotechnology [1,2,3,4]. However, the reproduction mechanism of nonmodel fish remains a less-than-comprehensive understanding due to various technical limitations. Fish, similar to other bisexually reproducing metazoans, also have two major cell lineages: soma cell and germ cell [5]. Among them, germ cells play an irreplaceable role in the reproduction of animals by producing gametes transmitting genetic information from one generation to the next [6].

The identification of marker genes in germ cells is an initiating step for studying their development. Germ cell development is crucial in organisms with sexual reproduction to complete their life cycle [7]. *Vasa* is one of the most studied universal markers and is used to identify the germ cells [8]. It is known as DEAD box polypeptide 4 (*Ddx4*), encoding the ATP-dependent RNA helicase, a crucial member of the DEAD-box family [9,10]. Similar to other members of the family, Vasa also contains nine conserved motifs, including an Asp-Glu-Ala-Asp motif—the origin of the family name [11]. The presence of these conserved domains enable them to exhibit meaningful functions in RNA from transcription to degradation [12]. Since it was first identified in fruit fly (*Drosophila melanogaster*) by mutagenesis [13], *vasa* homologs have been identified in many species, such as human (*Homo sapiens*) [14], mouse (*Mus musculus*) [15], tropical clawed frog (*Xenopus laevis*) [16], zebrafish (*Danio rerio*) [17] and amphipods (*Parhyale hawaiensis*) [18]. With a variety of *vasa* homologs having been characterized, evidence showed that the *vasa* gene is conserved among species [19].

Although *vasa* was widely defined as germ cell marker gene in many animals, their expression patterns vary and present species-specificity to a variable extent in different species [20]. In addition to their important roles as a germ cell marker, *vasa* can also play important roles in germ cell development, proliferation and differentiation; initially, it was identified to be responsible for the cause of failure in the formation of PGCs [21]. In *Drosophila*, *vasa* are expressed at two different stages: in embryos, corresponding to a maternal expression for the localization of the cytoplasmic determinants of the germline, and later during early oogenesis and spermatogenesis, for the development of oocytes and spermatocytes [22]. In addition, the intracellular localization of Vasa protein is closely associated with the chromatoid body during spermatogenesis [23]. The knockdown of *vasa* in zebrafish did not affect the establishment of the germline, but the disruption resulted in sterility in males [24,25]. Similarly, morpholino-silenced expression of medaka *vasa* demonstrated that *vasa* was required for migration but not survival of primordial germ cells [26]. Further, *vasa* also exerts a part in regulating the cell cycle, tumorigenesis and development [27,28,29].

Leopard coral grouper, *Plectropomus leopardus* is a commercially important marine fish, inhabiting the Great Barrier Reef [30]. Similar to most other groupers, it is a protogynous hermaphrodite, which begins as a female and later may switch to male [31,32]. This asynchronous maturation of males and females posed some obstacles to the artificial reproduction of fry [33]. For accelerating the process of artificial breeding, research on its reproductive and developmental mechanisms is necessary [5,34]. However, the underlying mechanism of germ cell development, sex differentiation and sex reversal for this specie is still far from being clarified. The discovery of specific germ cell marker genes may be of great help in the study of this process. In the present study, the *vasa* homolog *Plvasa* was identified and isolated from leopard coral grouper for the first time, and the expression and distribution of its mRNA and protein during gametogenesis in leopard coral grouper was examined. These results will provide evidence for further studies on the regulatory mechanisms of differentiation and migration, gonadal development and sexual differentiation of leopard coral grouper.

## 2. Materials and Methods

### 2.1. Animals and Ethics

Leopard coral groupers used in the study were obtained from Hainan Chen hai aquaculture Co., Ltd., in Dongfang, Hainan, China. Samples including heart, liver, spleen, kidney, muscle, intestine, brain, gill and gonad were collected randomly from six healthy adults after anesthesia with 0.05% solution of ethyl 3-aminoben-zoate methanesulfonate (Sigma-Aldrich, Shanghai, China). Each sample was collected in triplicate, and then frozen in liquid nitrogen and stored in an ultralow temperature refrigerator at −80 °C for RNA extraction. All procedures of the research were endorsed by complied with the guidelines and regulations established by the College of Marine Life Sciences, Ocean University of China Institutional Animal Care and Use Committee on 10 October 2018 (Project Identification Code: 20181010).

### 2.2. RNA Extraction and cDNA Synthesis

Total RNA from leopard coral grouper was extracted using TRIzol Reagent (Invitrogen, Carlsbad, CA, USA) and digested with DNase I (TaKaRa, Shiga, Japan) to remove potential DNA contamination. RNA concentration and purity were determined by NanoDrop One (Thermo, Waltham, MA, USA), and RNA integrity was verified by agarose gel electrophoresis. Only RNA samples with clear bands corresponding to 18S and 28S rRNA on the gel, an OD260/OD280 ratio between 1.8 and 2.0, and an OD260/OD230 ratio higher than 2.0 were used for subsequent experiments, and then frozen at −80 °C. The first-strand cDNA was synthesized by using 1 μg total RNA, random primers and Reverse Transcriptase M-MLV (RNase H-) Kit (TaKaRa, Shiga, Japan) [35].

### 2.3. Phylogenetic Analysis

The leopard coral grouper sequence in this research was obtained from CNGB under the accession CNA0007316 (https://db.cngb.org/, accessed on 30 May 2021), and the accession number in NCBI is NC_056486.1 (http://www.ncbi.nlm.gov, accessed on 2 June 2021). The specific primers Vasa-PCR-Fw and Vasa-PCR-Rv (Table 1) of *Plvasa* were designed for sequence verification. The BLAST search was applied to confirm the homologous nucleotide and protein sequences at the National Center for Biotechnology Information website (NCBI). Amino acid sequences of Vasa in 26 other species were downloaded from Ensemble (https://asia.ensembl.org, accessed on 2 June 2021) and NCBI (Table S1 in Supplementary Materials), including human, mouse, chimpanzee (*Pan troglodytes*), platypus (*Ornithorhynchus anatinus*), African clawed frog (*Xenopus tropicalis*) and other species. The homologous alignment of the coding sequences was produced with ClustalW and multialigned data was managed using the GENEDOC program [36]. In reconstructing the phylogenetic tree of the Vasa protein, the MEGA7 program carried out data processing by choosing neighbor-joining (NJ) with a bootstrap of 1000 replicates for the reliability test [37].

### 2.4. Synteny Analysis

The synteny and conservation of the *vasa* gene were acquired from the Genomics browser (https://www.genomicus.bio.ens.psl.eu/genomicus, accessed on 15 July 2021) [38]. Beyond that, annotated upstream and downstream genes surrounding *vasa* genes were collected from the genome databases at the NCBI. According to the position and relative locations of these genes on the chromosome or linkage group, these genes were mapped along the chromosome axes by synteny analysis.

### 2.5. Genomic Structure Analysis

The splicing sites of exons and introns, together with their positions and sizes in the genome, were identified according to the sequencing results of *vasa* mRNA and the full genome of leopard coral groupers [39]. The SMART website (https://smart.embl.de, accessed on 27 August 2021) was used for the identification and annotation of protein domains and the analysis of protein domain architectures [40]. IBS (Illustrator for Biological Sequences) was applied in drawing structure diagrams of nucleotide sequences with these data.

### 2.6. Quantitative Real-Time PCR (qPCR)

Following the principle of primer design, pairs of *Plvasa* primers (Table 1) were designed based on sequence information from the Integrated DNA Technologies website (http://sg.idtdna.com/Primerquest/Home/Index, accessed on 3 May 2021). The *Rpl13* and *B2m* were used as double internal reference genes [41]. The assays were performed on RocheLightCycler480 (Roche Applied Science, Mannheim, Germany) and the qPCR reaction system (20 µL), containing 10 μL 2 × SYBR qPCR Master Mix (US Everbright Inc., Suzhou, China), 0.4 μL of each primer (10 μM), 1 μL diluted cDNA (20 ng/μL) and 8.2 μL nuclease-free water. The cycling program was initiated from 95 °C (5 min) for preincubation, followed by 35 cycles at 95 °C (15 s) and 60 °C (45 s). Each group of reactions was performed with three biological replicates and three technical replicates. Fusion curve analysis was added after each PCR procedure to ensure the specificity of quantitative PCR amplification. The relative expression of the *vasa* gene was calculated by the 2^−ΔΔCt^ comparative Ct method. SPSS 20.0 was used for one-way ANOVA analysis and graph-based posterior probability tests; *p* < 0.05 represented significant difference.

### 2.7. Fluorescence In Situ Hybridization (FISH)

The protocols followed the methods described in a previous study [42], with a few modifications as detailed below. Vasa-ISH-Fw/Rv specific primers (Table 1) were designed for acquiring the fragment of Plvasa. The fragment was amplified by PCR and cloned into pMD^TM^-19T Vector (TaKaRa, Shiga, Japan). Sense and antisense RNA probes were synthesized by in vitro transcription from the plasmid clone under the drive of the T7 or SP6 promoter with the DIG RNA Labeling Mixture (Roche, Mannheim, Germany). The gonad tissues from ten randomly selected healthy 6-month-old and adult fish were fixed in 4% paraformaldehyde overnight and embedded in paraffin after dehydration by ethanol series and clearance by xylene. The hematoxylin and eosin (H&E) stained sections were observed to find the suitable position for FISH. Paraffin-embedded gonad samples were sectioned with a thickness of 6 μm and 12 slices were randomly collected from each sample; after rehydration and dewaxing, the gonad sections were digested with 2 μg/mL proteinase K at 37 °C for 15 min. After prehybridization at 56 °C for 4 h, hybridization was performed with 1 ng/μL denatured RNA probe in hybridization buffer at 56 °C for 16 h. Then, the probes were washed away, and antibody incubation was performed in a fresh solution of Anti-digoxigenin-fluorescein at 37 °C for 1 h. Nuclei were stained with DAPI, and slides were mounted with the antifade mounting medium (Beyotime, Shanghai, China). The signals were taken under a confocal laser microscope (OLYMPUS, Tokyo, Japan).

### 2.8. Immunohistochemistry (IHC)

Based on the previous studies [43], gonad tissues from six randomly selected healthy adult fish were fixed in 4% paraformaldehyde (Sigma-Aldrich, Saint Louis, MO, USA) overnight, dehydrated in increasing concentrations of ethyl alcohol, cleared with xylene, and then embedded in paraffin. More than 12 slices of 6 µm thickness per sample were cut for analysis with a microtome. Rehydrated sections were treated to inactivate endogenous enzymes with 3% H_2_O_2_ for 10 min and washed 3 times in PBS. The sections were heated at 97 °C in 0.01 M trisodium citrate buffer for repairing the antigen, and then cooled to room temperature, and washed 3 times in PBS. Bovine serum albumin (BSA: PBS = 1:10) solution was used for blocking nonspecific binding sites. The tissues were incubated overnight at 4 °C with the rabbit anti-Vasa antibody (1:200 dilution with 1% goat serum in PBS), and then incubated at room temperature for 1 h with the second antibody, Biotin-Goat Anti-Rabbit IgG at a dilution of 1:1000 with 1% goat serum in PBS. Two incubation processes were carried out successively. Importantly, it should be thoroughly washed with PBS before performing the next step. The sections were developed with DAB-H_2_O_2_ and stained again with hematoxylin. Finally, the neutral resin was added to seal the sections. An OLYMPUS biological microscope (OLYMPUS, Tokyo, Japan) was used to observe and photograph the results.

### 2.9. Density Gradient Centrifugation and qPCR Analysis

After the fish surface was disinfected with 75% ethanol, the gonad tissues were excised aseptically from 6-month-old juvenile leopard coral groupers. All minced gonads were lysed by protease digestion solution (0.25% trypsin, 2.0 mg/mL collagenase H, 5.0% FBS and 0.05% DNase I dissolved in L-15 Medium (Leibovitz) for 1.5 h at 28 °C). The dissociated cell suspension was filtered through the cell strainer (nylon mesh, Biosharp), and then all single cells were collected by centrifugation at 100× *g* for 10 min and relevitated in the culture medium. The cell suspension was separated by Percoll density gradient centrifugation (20%, 30%, 40%, 50% and 65% Percoll in saline solution, SS) for 25 min at 400× *g*. After centrifugation, the cell layers were generated in different concentrations of Percoll. The cells in each layer were observed under a microscope and identified by measuring the cell diameter. RNA from each cell layer was extracted using TRIzol Reagent separately and reversed transcribed into cDNA following the steps above. *Plvasa* primers were used for gene expression study for the different layers of cells by qPCR.

## 3. Results

### 3.1. Phylogenetic Analysis of Vasa Genes

**Figure 1:**
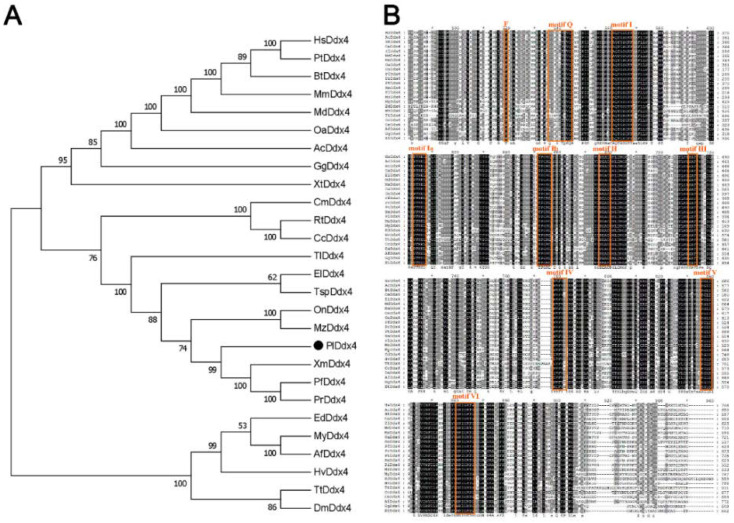
Sequence alignment and phylogenetic tree of *vasa* genes. (**A**) Phylogenetic tree of Vasa from leopard coral grouper and other species. The phylogenetic relationship of Vasa protein was inferred with the neighbor–joining method by MEGA7. All the assembly ID of *vasa* homologs can be found in Table S1 from NCBI, Ensemble, and UniProt. Mammals—HS: *Homo sapiens*; Pt: *Pan troglodytes*; Bt: *Bos taurus*; Mm: *Mus musculus*; Md: *Monodelphis domestica*; Oa: *Ornithorhynchus anatinus*; Reptiles—Ac: *Anolis carolinensis*; Birds—Gg: *Gallus gallus*; Amphibias, Xt: *Xenopus tropicalis*; Fishs—Cm: *Callorhinchus milii*; Rt: *Rhincodon typus*; Cc: *Carcharodon carcharias*; Tl: *Tetraodon Linnaeus*; El: *Esox Lucius*; Tsp: *Threespine stickleback picornavirus*; On: *Oreochromis niloticus*; Mz: *Maylandia zebra*; Pl: *Plectropomus leopardus*; Xm: *Xiphophorus maculatus*; Pf: *Poecilia Formosa*; Pt: *Poecilia reticulata*; Invertebrates—Ed: *Exaiptasia diaphana*; My: *Mizuhopecten yessoensis*; Af: *Azumapecten farreri*; Hy: *Hydra vulgaris*; Tt: *Trichuris trichiura*; Dm: *Drosophila melanogaster*. (**B**) Multiple alignments of the *vasa* between leopard coral grouper and other species. The ClustalW performed multiple sequence alignment and the GENEDOC viewed and managed the aligned data. The shade of color indicate the degree of conservatism. Orange blocks indicate the ten conserved regions of the DEAD-box protein family, including motif Q (GYTKLTPVQ), motif I (AQTGSGKT), motif Ia (PTREL), motif Ib (TPGRL), motif II (DEAD), motif III (SAT), motif IV (MVFVE), motif V (RGLD) and motif VI (HRIGRTG).

The phylogenetic tree was constructed using the N–J algorithm, which demonstrated the evolutionary relationship of Vasa (Figure 1A). The generated bootstrap consensus tree displayed three distinct branches: tetrapods, fish and other clusters containing several invertebrates. The result supported the early evolution and divergence of the *vasa* gene before fish and tetrapod separation. Fish were separated from the other two branches and divided into two categories: teleost and cartilaginous fish. *Plvasa* were close to *vasa* branches of other teleosts, such as southern platyfish (*Xiphophorus maculatus*) and guppy (*Poecilia reticulata*), which was consistent with cognition of the taxonomic relationship.

The amino acid sequence alignment of Vasa protein demonstrated all the species contained the typical and conserved structural characteristics of the DEAD-box protein family nine consensus motifs characteristic of the DEAD-box protein family from invertebrates and vertebrates (Figure 1B). The nine conserved motifs were found by using BLAST against the Conserved Domain Database, including motif Q (GYTKLTPVQ), motif I (AQTGSGKT), motif Ia (PTREL), motif Ib (TPGRL), motif II (DEAD), motif III (SAT), motif IV (MVFVE), motif V (RGLD) and motif VI (HRIGRTG).

### 3.2. Gene Structure Analysis of Vasa Genes

**Figure 2:**
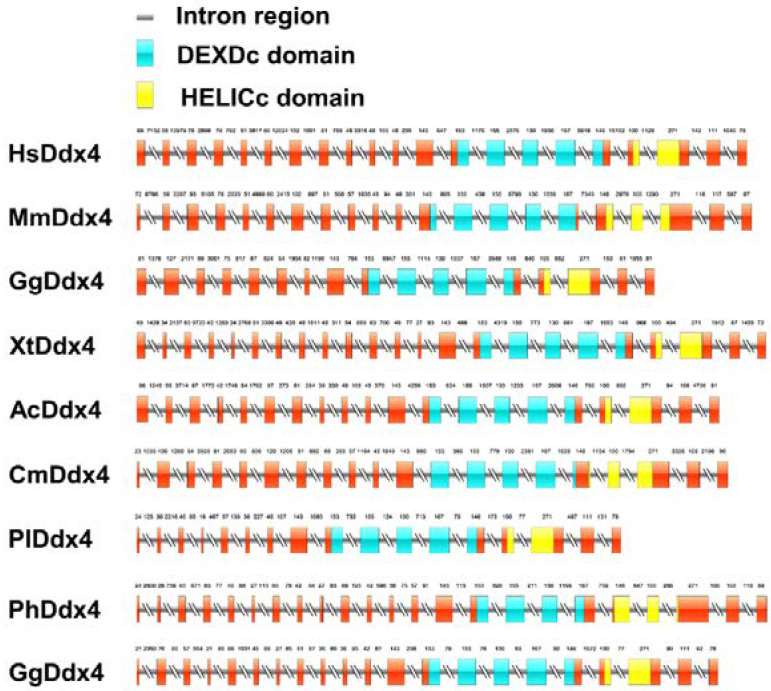
Schematic genomic structure of *vasa* genes between leopard coral grouper (*Plectropomus leopardus*) and other vertebrates. Red, blue and yellow rectangles represent exons: blue blocks indicate the DEXDc domain and yellow blocks indicate the HELICc domain. Rectangles with double slash represent introns. DEXDc, DEAD-like helicases superfamily; HELICc, helicase superfamily C-terminal.

The differences in genomic structure in selected species are shown in Figure 2. The full length of *Plvasa* cDNA was 8643 base pairs (bp). As the graph exhibits the number of coding exons that varied among species, the shortest was 17 exons, and the longest was 23 exons. Among them, eight exons were found to have the same size and position, which showed good conservatism. Additionally, two main domains of Vasa—Domain I (DEADc domain, DEAD-like helicases superfamily) and Domain II (HELICc domain, helicase superfamily C-terminal)—are located in the region where the protein sequence conservatism is higher. Previously, studies have shown that HELICc was sufficient for germ-plasm localization by interacting with Oskar (Osk) [44].

### 3.3. Synteny Analyses of Vasa Genes

**Figure 3:**
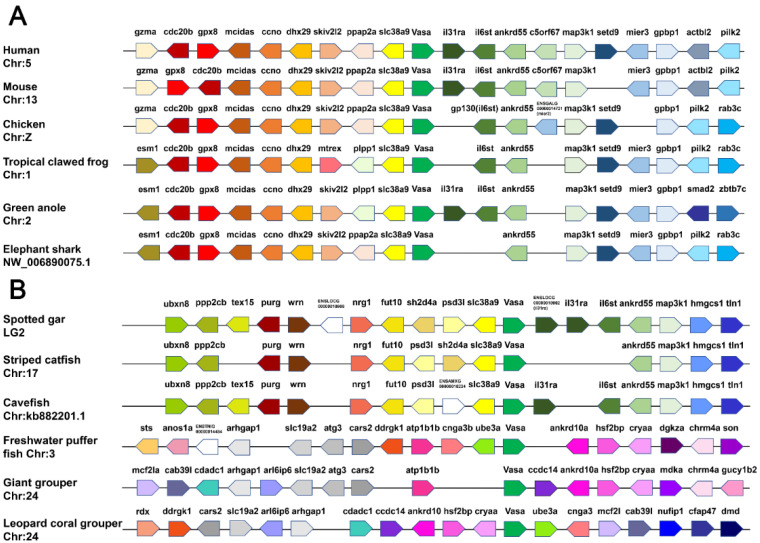
Synteny analyses of *vasa* genes in vertebrates. (**A**) Chromosomal segments showing the conserved syntenic block around *vasa* are shown from human, mouse, chicken, tropical clawed frog, green anole and elephant shark. (**B**) The syntenic blocks of *vasa* in teleosts, including spotted gar, striped catfish, cavefish, freshwater puffer fish, giant grouper and leopard coral grouper (*Plectropomus leopardus*). Different genes are marked with different colored arrows. The direction and position of the arrows indicate the genomic direction and position in the same chromosome or scaffold, respectively, relative to several other genes. The empty spaces indicate regions with the absence of the gene in the genome.

As shown in Figure 3, *vasa* and the adjoining genes were mapped according to their relative locations on the same scaffold or chromosome. The upstream and downstream genes adjacent to *vasa* were highly conserved and shared a common direction in mammals, amphibians and birds along the chromosomes, whereas, there were two different modes in teleost. Firstly, we found that *vasa* adjoining genes shared relatively highly conserved synteny in spotted gar (*Lepisosteus oculatus*), striped catfish (*Pangasianodon hypophthalmus*) and cavefish (*Amblyopsis spelaea*). The upstream (*ppp2cb*, *purg*, *wrn*, *nrg1*, *fut10* and *slc38a9*) and downstream (*il31ra*, *il6st*, *ankrd55*, *map3k1*, *hmgcs1* and *tln1*) genes shared the same direction in different species, roughly. The second mode was that the genes surrounding *vasa* in the three teleosts—freshwater puffer fish (*Tetraodon Linnaeus*), giant grouper (*Epinephelus lanceolatus*) and leopard coral grouper were completely different from those described above, and many genes that existed in the aforementioned teleost were lost, such as *slc38a9*, *il31ra*, *il6st*, *ankrd55* and *map3k1*. The synteny relationships in the three teleosts were not consistent, but their synteny gene arrangement showed certain regularity. As shown in Figure 3, *Plvasa* was found on leopard coral grouper chromosome 24, and the inversion, shearing and recombination existed in the upstream and downstream regions compared with those in the other two fish during evolution. The genes *ddrgk1*, *cnga3* and *mcf21* have undergone complex rearrangements, and *ankrd10a*, *hsf2bp* and *cryaa* were conserved with the corresponding regions.

### 3.4. Gonad-Specific Expression of Vasa in Leopard Coral Grouper

**Figure 4:**
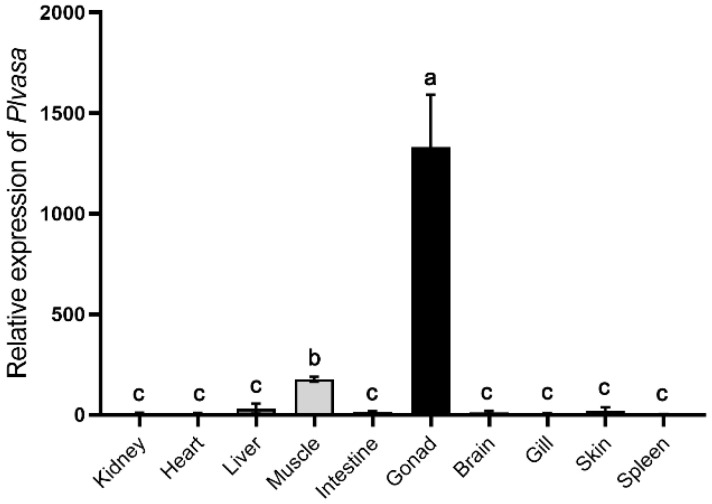
Analysis of *Plvasa* expression patterns at the level of mRNA in gonads of leopard coral grouper (*Plectropomus leopardus*) by qRT-PCR. The *rpl13* and *b2m* were used as double internal reference genes to exhibit the expression level of *Plvasa* mRNA in different tissues, including kidney, heart, liver, muscle, intestine, gonad, brain, gill, skin and spleen. Relative expression was calculated by the 2^−ΔΔCt^ method. Data are shown as mean ± SD. Letters (a, b and c) reveal statistical significance (*p* < 0.05), which was calculated by one-way analysis of variance.

Tissue distribution patterns of *Plvasa* mRNA were analyzed in kidney, heart, liver, muscle, intestine, gonad, brain, gill, skin and spleen of leopard coral grouper (Figure 4). The expression levels of the *Plvasa* gene varied significantly among tissues in individuals. The expression level of mRNA transcript was high in gonads and significantly higher than in other tissues. Additionally, the expression of *vasa* mRNA is also detected in muscle and significantly lower than that in gonads, while the expression was barely detected in other tissues (Figure 4).

### 3.5. Germline Cell-Specific Expression of Vasa in Leopard Coral Grouper

**Figure 5:**
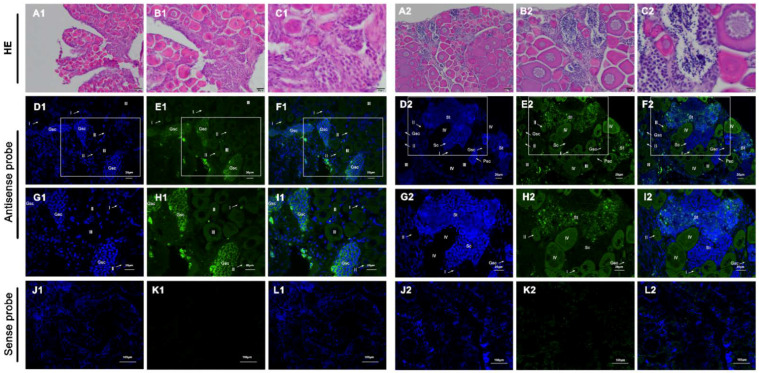
Localization of *Plvasa* RNA by fluorescence in situ hybridization (FISH) and histology. (**A1**–**L1**,**A2**–**L2**) were 6-month-old and adult (with intersexual gonads) fish, respectively. (**A1**–**C1**,**A2**–**C2**) were stained with H&E ((**A**): 20×, (**B**): 40× and (**C**): 100×). Gonadal sections were stained (**E**,**H**,**K**) green for *vasa* with digoxigenin (DIG) and (**D**,**G**,**J**) blue for DNA with diamidino-phenyl-indole (DAPI); merged images (**F**,**I**,**L**). The box indicates the area magnified in the next frame ((**D**–**F**,**J**–**L**): 40×; (**G**–**I**): 60×). Positive signals with antisense probe hybridization are shown in (**D**–**I**). No staining was seen with the sense probe in (**J**–**L**). Staining was analyzed by confocal microscopy. Gsc: germ stem cells; I, early previtellogenic phase; II, late previtellogenic phase; III, early vitellogenic phase; IV, fully vitellogenic phase; Psc: primary spermatocyte phase; Sc, spermatocytes; St, spermatids.

The anatomical (Figure S1) and hematoxylin and eosin (H&E) staining showed that the gonads of 6-month-old fish had a certain number of germ stem cell and primary oocytes, and the gonads of adult fish in an intersexual period, containing both male and female germ cells. According to the specific expression of *vasa* mRNA, its distribution in gonads of 6-month-old and adult (with intersexual gonads) fish was detected by fluorescence in situ hybridization (FISH) with digoxigenin (DIG)-labeled antisense RNA probe. The gonads of 6-month-old fish were in the female stage of development, and the expression of *vasa* mRNA was highest when the germ cells were at germline stem cells. The *vasa* mRNA was abundantly expressed in germline stem cells, moderately expressed in oocytes of stage I, stage II and stage III, and absent in somatic cells. In gonads of the intersexual development stage, *Plvasa* mRNA positive signals were observed in both female and male germ cells, and no signal was detected in the somatic cells (Figure 5). Further, we observed that spermatocytes and spermatids were located around the female germ cells and distributed throughout the gonads. In male germ cells, the hybridization signals were the most intense in spermatids and became weak in primary spermatocytes, and weaker in spermatocytes. In female germ cells, oocytes in the four stages were observed. Stage I and II were previtellogenic, stage III was vitellogenin, and stage IV was fully vitellogenin at which time the oocytes were maturing. The fluorescence signal of *Plvasa* was distributed in germline stem cells and oocytes at various stages throughout the cytoplasm. With the development of oocytes, the signals become weak until they were difficult to detect.

**Figure 6:**
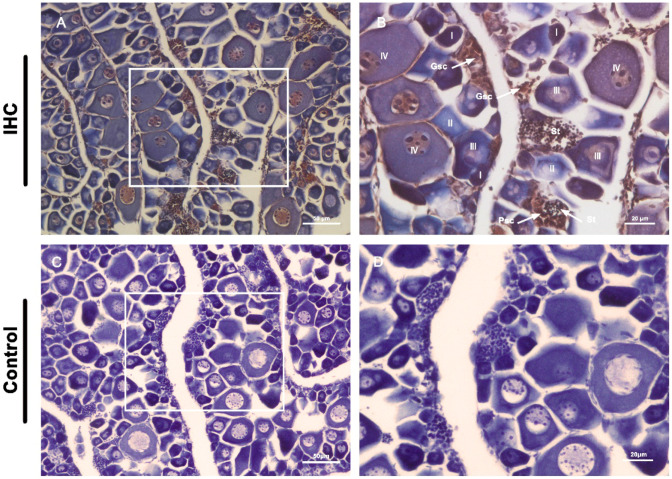
Localization of Plvasa protein by immunohistochemistry (IHC) in intersexual gonads of adult leopard coral grouper (*Plectropomus leopardus*). Positive signals of anti-Vasa immunolabeling are shown in tawny (**A**,**B**). Control groups are shown in (**C**,**D**). The box indicates the area magnified in the next frame ((**A**,**C**): 20×; (**B**,**D**): 40×). Gsc: germ stem cells; I, early previtellogenic phase; II, late previtellogenic phase; III, early vitellogenic phase; IV, fully vitellogenic phase; Psc: primary spermatocyte phase; Sc, spermatocytes; St, spermatids.

Next, the distribution of Vasa protein was further determined by immunohistochemical analysis (Figure 6). IHC results showed that the intersexual gonads were composed of large numbers of oocytes and spermatocytes in different development stages. As shown in Figure 6, the signals of Vasa protein were also observed throughout oogenesis, with the highest in the germline stem cells and strong in oocytes at previtellogenic and early vitellogenin stages. The cytoplasm of mature oocytes appeared mauve and the individual cells were relatively large. The expression of Vasa protein was also observed in primary spermatocytes and spermatids, while they were not detected in the somatic cells. The results of the analysis revealed that all germ cells had cytoplasmic Vasa protein and the expression varied with cell maturity.

### 3.6. Density Gradient Centrifugation Enrichment and Relative Expression of Vasa in Different Types

**Figure 7:**
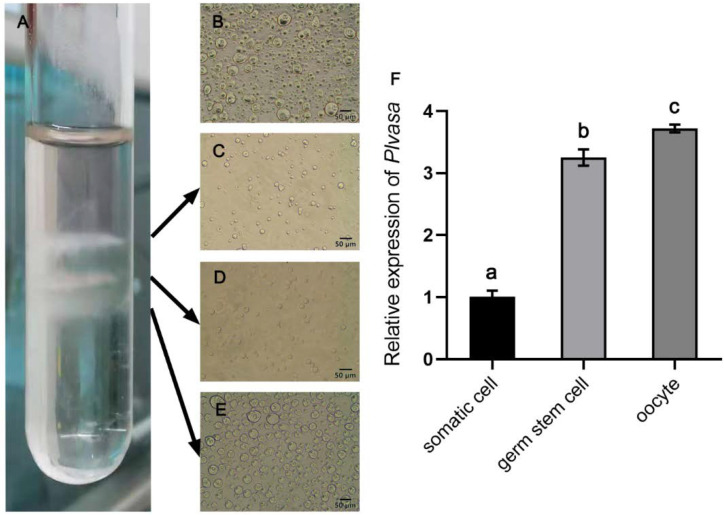
Purification enrichment of germ stem cells from 6-month-old leopard coral grouper (*Plectropomus leopardus*) by Percoll centrifugation and expression levels of *plvasa*. (**A**) Three layers in the tube after purification. The gradient-separated cells in three layers were observed and detected individually. (**B**) The results of cell dissociation before purification. (**C**) The cells present in the first layer contained a higher percentage of somatic cells. (**D**) Germ stem cells in the second layer. (**E**) The oocytes were at different stages in the third layer. (**F**) Expression levels of *vasa* in purified cells of three layers by q-PCR. The relative expression of each layer (somatic cells, germ stem cells and oocytes) was calculated by the 2^−ΔΔCt^ method. Data are shown as mean ± SD. Letters (a, b and c) reveal statistical significance (*p* < 0.05), which was calculated by one-way analysis of variance.

The dissociated cell suspension from the gonad of leopard coral groupers was separated into three layers using Percoll density gradient centrifugation (Figure 7A). The first layer was intensely thin and not easily observed, which mainly contained somatic cells (Figure 7C). The second layer contained large numbers of germline stem cells (large round cells, spherical nuclei, transparent cytoplasm and high nucleus/cytoplasm ratio). The proportion of germ stem cells was significantly higher than that in dissociated cells from gonads (Figure 7B). In the third layer (Figure 7D), most of the cells were oocytes and cell clusters also could be observed under phase-contrast microscopy. Further, the expression of Plvasa in each layer showed that the transcript level was high in germline cells and almost undetectable in somatic cells (Figure 7F), and the oocyte showed the highest expression level, which was significantly higher than the germ stem cells.

## 4. Discussion

This study reports the isolation and characterization of *vasa* homolog in *Plectropomus leopardus*. Multiple sequence alignments exhibited the *Plvasa* possessed representatively conserved motifs of the DEAD-box family that have been identified in other species, such as tammar (*Macropus eugenii*), platypus and brown-marbled grouper (*Epinephelus fuscoguttatus*) [45,46]. These motifs play a crucial role in ATP binding, RNA binding and interdomain interactions in many species [47,48]. The function of Vasa is determined by motifs together with two larger functional units (domain)—DEADc domain and HELICc domain—that were ubiquitous in the species we analyzed. Studies have shown that these two series-wound and repeated RecA-like domains are core features in the region for hydrolyzing ATP and driving RNA duplex unwinding, with N-terminal RecA-like domain (NTD) and C-terminal domain (CTD) [44,47]. These suggest that our leopard coral grouper sequence we characterized encodes a protein member of the DEAD-box family with an ATP-dependent RNA helicase function.

The results of phylogenetic reconstruction and genomic synteny analyses support that *Plvasa* is a true *vasa* homolog. In the phylogenetic tree, the Vasa of leopard coral grouper was clustered into the teleost branch and separated from other fish, which showed their genetic relationship was very close. The phylogenetic relationship was in accord with the traditional relatedness classification [49]. The whole-genome duplication (WGD) has been implicated in the evolution of teleost fishes [50]. Previous studies showed that most teleost-specific whole-genome duplication (TSWGD) duplicates gained their current status (loss of one duplicate gene or retention of both duplicates) relatively rapidly after TSWGD [51]. The genomic synteny analyses showed that the *atg3* gene in leopard coral grouper was lost, and the segments contained in *ankrd10a*, *hsf2bp* and *cryaa* occurred through chromosomal translocations, compared to freshwater puffer fish and giant grouper. It could be reasonably inferred that the TSWGD events have shaped the result of the alteration of the gene near *Plvasa* [52].

Previously, the expression and localization of *vasa* have been studied in many species by various methods such as RT-PCR, in situ hybridization and immunohistochemistry [15,53,54,55]. In the present study, we identified the expression of *Plvasa*, and the result showed that it was highly detected in gonads, lowly in muscle and barely in other tissues (Figure 4). Similarly, *vasa* was also detected to be weakly expressed in muscle of tongue sole (*Cynoglossus semilaevis*), which may be related to its helicase activity [56,57]. During spermatogenesis and oogenesis, the expression patterns of *vasa* are species-specific but still have some commonality in many species [58,59]. In oogenesis, the detected expression levels of *vasa* in most species were low in the early stage, the highest in oogonia and primary oocytes, and after that gradually decreased [60,61,62]. In spermatogenesis, *vasa* expression was strongest in spermatogonia, weakened in spermatocytes and not detected in spermatids [63,64]. In leopard coral grouper, *Plvasa* mRNA and protein showed strong expression levels in spermatids, germline stem cells and oocytes at I–II stages. The expression results at the mRNA level were similar to the Chinese soft-shell turtle (*Pelidiscus sinensis*), which were found to be weak in spermatogonia and strong in spermatids [20]. Moreover, the *Plvasa* mRNA and protein seemed predominantly uniform in the cytoplasm, while those of other teleosts were generally spread uniformly in the perinuclear area or formed distinct patches in the cytoplasm [19,61,65]. This is similar to the results in the early developing oocytes of Chinese sturgeon (*Acipenser sinensis*) and may be attributed to the fact that mitochondrial clouds are distributed throughout the cytoplasm and *vasa* is usually colocalized with mitochondrial clouds [64,66]. The possibility also exists that *Plvasa* has this distribution characteristic in lately stage oocytes. All of the results indicated that *vasa* as a maternal effector gene [53] might play an important role in germ cells’ differentiation of leopard coral grouper.

As previous studies in the teleost, the *Plvasa* gene is also specifically expressed in germline cells, which might become an important molecular marker to explore the processes of germ cell generation, migration and differentiation. Germ cells segregate from the early embryonic cells and migrate toward the genital ridge, and then produce gametes in the gonad through meiosis [67]. The search for molecular marker genes that are specifically expressed in germ cells is crucial to the study of gametogenesis. *Vasa*, as a maternal-effect gene of the reproductive line, is very important for gametogenesis. In teleosts, *vasa* is a useful marker for exploring gonad development and early gametogenesis. It has been identified as a reproductive marker in Asian seabass (*Lates calcarifer*), tambaqui (*Colossoma macropomum*) and rare minnow (*Gobiocypris rarus*) [61,65,68]. Additionally, the chimera mRNA of *vasa*-3′UTR can trace PGCs in zebrafish, rainbow trout (*Oncorhynchus mykiss*), Nibe croaker (*Nibea mitsukurii*), tongue sole (*Cynoglossus semilaevis*) among others [69,70]. The findings provide molecular evidence for further studying the germ differentiation pattern of leopard coral grouper. Furthermore, the analysis of its gene structure and synteny with upstream and downstream genes can also provide a strong theoretical basis for the further study and manipulation of reproductive regulation in leopard coral grouper.

## 5. Conclusions

In conclusion, we found a homolog of *vasa* in hermaphroditic leopard coral grouper and identified mRNA characteristically expressed in the gonad tissues. It is the first report to identify *Plvasa* as a germ cell marker of the leopard coral grouper that can be used to track the entire process of germ cell development, providing us with basic tools for our understanding of germline development in leopard coral grouper.

## Figures and Tables

**Table 1:** Sequences of primers used in the study.

Purpose	5′ to 3′ Sequence
Vasa-PCR-Fw	ATGGACGAGTGGGATGAA
Vasa-PCR-Rv	CACCGTTGTCCTGGAAAG
Vasa-qRT-PCR-Fw	AAGACAGACTACTTGTTCTTGGCTG
Vasa-qRT-PCR-Rv	CAAGGAGCTGATCTCTCTTGCA
Vasa-ISH-Fw	ATTTAGGTGACACTATAGCTGATTTCCTCGCCGCTT
Vasa-ISH-Rv	TAATACGACTCACTATAGGGTGGCTCTTCACACCGTTGTC
Rpl13-qRT-PCR-Fw	CAGCGTCTGAAGGAGTACCG
Rpl13-qRT-PCR-Rv	ACCAGCGAGCTGAGTGGC
B2m-qRT-PCR-Fw	GGCAATTCCACCTGACCAAG
B2m-qRT-PCR-Rv	CAACCCAGGCATATTCCTTAACT

## Data Availability

Not applicable.

## Supplementary Materials

The following supporting information can be downloaded at: https://www.mdpi.com/article/10.3390/genes13061077/s1, Table S1: Database ID of the sequences used in this study. Figure S1: The gonadal anatomy of leopard coral grouper (*Plectropomus leopardus*).
